# Author Correction: A transcriptomic atlas of mouse cerebellar cortex comprehensively defines cell types

**DOI:** 10.1038/s41586-021-04373-7

**Published:** 2022-01-12

**Authors:** Velina Kozareva, Caroline Martin, Tomas Osorno, Stephanie Rudolph, Chong Guo, Charles Vanderburg, Naeem Nadaf, Aviv Regev, Wade G. Regehr, Evan Macosko

**Affiliations:** 1 Broad Institute of Harvard and MIT, Stanley Center for Psychiatric Research, Cambridge, MA USA; 2 Department of Neurobiology, Harvard Medical School, Boston, MA USA; 3 Department of Psychiatry, Massachusetts General Hospital, Boston, MA USA

**Keywords:** Genomics, Cellular neuroscience

Correction to: *Nature* 10.1038/s41586-021-03220-zPublished online 6 October 2021

In the version of this article initially published, there was a typo in the Data availability section. The Gene Expression Omnibus accession has been updated to GSE165371.

The changes have been made in the online version of the paper.

