# Supplementary material for: A transcriptomic atlas of mouse cerebellar cortex comprehensively defines cell types
Source: Nature. 2021 Oct 6;598(7879):214–9. doi: 10.1038/s41586-021-03220-z (PMC8494635; doi:10.1038/s41586-021-03220-z)
Supplement: Supplementary file 1 — This file contains Supplementary Note for Extended Data Fig. 8, legends for Supplementary Tables 1-4, and Supplementary References. [file 41586_2021_3220_MOESM1_ESM.pdf]

nature portfolio

https://doi.org/10.1038/s41586-021-03220-z

Supplementary information

A transcriptomic atlas of mouse cerebellar cortex comprehensively defines cell types

In the format provided by the authors and unedited

Supplementary Information

Supplementary note for Extended Data Fig 8:

Previous studies indicate that there is a general trend for spikelets being more prevalent in the inner third than in the outer third of the molecular layer. This is consistent with estimates from studies in young rats and mice of electrical coupling and connexin 36 staining that suggest more prevalent coupling in the inner third than in the outer third[1]. The lack of spikelets in a cell could reflect no gap junction coupling, a lack of activity in neighboring MLIs, or death of neighboring cells during the slicing procedure. Our recordings of MLIs indicate that the vast majority of MLI1 and MLI2 neurons are spontaneously active (Fig. 4g,h), so that is unlikely to be a contributing factor to a lack of spikelets in a cell. Together the properties in Fig. 4 and in this figure suggest that MLI1s and MLI2s in the inner third of the molecular layer can be identified based on their electrical properties, with MLI1 having a lower R_m, likely having spikelets, having a small I_H, and having a lower capacitance. It is more difficult to discriminate between MLI1 and MLI2 neurons in the distal third of the molecular layer based solely on electrical properties.

Supplementary Table Legends

Supplementary Table 1: Established cell type markers. Markers from existing studies used to assign clusters to specific established cerebellar cell type identities. Genes that lack a citation were used in annotation by examining the spatial localization in the AIBA[2]. Note that globular and candelabrum types could not be disentangled based upon the review of markers in the existing literature.

Supplementary Table 2: Highly differentially expressed genes across all clusters. Genes are limited to top 500 differentially expressed genes per subtype. Column names represent the following: “avgExpr” is the mean gene expression in that subtype; “logFC” is the log fold change between cells within the subtype compared to remaining cells; “pval” is the p-value, calculated with the two-tailed Gaussian approximation of U statistic; “padj” is the Benjamini-Hochberg adjusted p-value; “pct_in” is the percent of cells in the subtype expressing the gene; “pct_out” is the percent of cells outside the subtype expressing the gene.

Supplementary Table 3: Summary of electrophysiological data. Number of cells recorded for the data in Fig 4 and Extended Data Fig 8. Statistical tests and p-values are reported for each experiment. For analysis of input-output curves, a Poisson generalized linear mixed effect model of the form SpikeCount ~ 1 + CurrentSteps + CellType + CurrentSteps*CellType + (1+CurrentSteps|CellIDs), with a canonical log link function, was fit using maximum pseudo-likelihood estimation.

Supplementary Table 4: Function parameters used during iterative cluster annotation. Parameters used for LIGER analyses and cluster annotation of major cell types. Parameters correspond to functions as follows: selectGenes: var.thresh; optimizeALS: k; makeSNN: dims.use; calcClusters: resolution. For each analysis round, clusters_retained and

clusters_removed are mutually exclusive so only one is indicated. Note that for some final annotations, clusters were merged based on shared expression of markers. For the Bergmann and Astrocyte annotations, the first round was performed on both populations at once to identify potential doublets, as Bergmann glia are a specialized population of astrocytes.

Supplementary References

1. Alcami, P. & Marty, A. Estimating functional connectivity in an electrically coupled interneuron network. *Proc. Natl. Acad. Sci. U. S. A.* **110**, E4798–807 (2013).
2. Lein, E. S. *et al.* Genome-wide atlas of gene expression in the adult mouse brain. *Nature* **445**, 168–176 (2007).
