# Supplementary material for: A transcriptomic atlas of mouse cerebellar cortex comprehensively defines cell types
Source: Nature. 2021 Oct 6;598(7879):214–9. doi: 10.1038/s41586-021-03220-z (PMC8494635; doi:10.1038/s41586-021-03220-z)
Supplement: Supplementary file 2 — Reporting Summary [file 41586_2021_3220_MOESM2_ESM.pdf]

nature research

Corresponding author(s): Evan Macosko

Last updated by author(s): December 4, 2020

Reporting Summary

Nature Research wishes to improve the reproducibility of the work that we publish. This form provides structure for consistency and transparency in reporting. For further information on Nature Research policies, see Authors & Referees and the Editorial Policy Checklist.

Statistics

For all statistical analyses, confirm that the following items are present in the figure legend, table legend, main text, or Methods section.

n/a	Confirmed	
☐	☒	The exact sample size (n) for each experimental group/condition, given as a discrete number and unit of measurement
☐	☒	A statement on whether measurements were taken from distinct samples or whether the same sample was measured repeatedly
☐	☒	The statistical test(s) used AND whether they are one- or two-sided<br>*Only common tests should be described solely by name; describe more complex techniques in the Methods section.*
☐	☒	A description of all covariates tested
☐	☒	A description of any assumptions or corrections, such as tests of normality and adjustment for multiple comparisons
☐	☒	A full description of the statistical parameters including central tendency (e.g. means) or other basic estimates (e.g. regression coefficient) AND variation (e.g. standard deviation) or associated estimates of uncertainty (e.g. confidence intervals)
☐	☒	For null hypothesis testing, the test statistic (e.g. F, t, r) with confidence intervals, effect sizes, degrees of freedom and P value noted<br>*Give P values as exact values whenever suitable.*
☒	☐	For Bayesian analysis, information on the choice of priors and Markov chain Monte Carlo settings
☒	☐	For hierarchical and complex designs, identification of the appropriate level for tests and full reporting of outcomes
☒	☐	Estimates of effect sizes (e.g. Cohen's d, Pearson's r), indicating how they were calculated

Our web collection on statistics for biologists contains articles on many of the points above.

Software and code

Policy information about availability of computer code

Data collection	Mouse raw sequencing data was processed, aligned and converted to digital gene expression matrix format by CellRanger v.3.0.2 (available from 10x Genomics) with default settings. Human raw sequencing data was processed, aligned and converted to digital gene expression matrix format using the open-source Drop-seq alignment workflow. ISH imaging data was acquired with Nikon NIS Elements v4.6. Two-photon imaging data was acquired with Scanimage on MATLAB R2009b. Electrophysiology data was acquired with Igor Pro 8 (Wavemetrics) and software mafPC (https://www.xufriedman.org/mafpc).
Data analysis	Data was analyzed using Seurat v2.3.4, LIGER v0.4.2, Monocle v2.10.1, on R v3.5.3, and Monocle3 v0.2.2 on R v3.6.3.,in addition to custom code and scripts available at https://github.com/MacoskoLab/cerebellum-atlas-analysis. Two-photon images were processed with ImageJ v2.0.0-rc-68/1.53e, and ISH imaging data was processed with ImageJ v2.0.0-rc-68/1.53e and Nikon NIS AR.

For manuscripts utilizing custom algorithms or software that are central to the research but not yet described in published literature, software must be made available to editors/reviewers. We strongly encourage code deposition in a community repository (e.g. GitHub). See the Nature Research guidelines for submitting code & software for further information.

Data

Policy information about availability of data

All manuscripts must include a data availability statement. This statement should provide the following information, where applicable:

- Accession codes, unique identifiers, or web links for publicly available datasets
- A list of figures that have associated raw data
- A description of any restrictions on data availability

All processed data and annotations have been made freely available for download and visualization through an interactive Single Cell Portal study (https://singlecell.broadinstitute.org/single_cell/study/SCP795/). Raw and processed data that support the findings of this study have been deposited in GEO under accession number XXX and in at the Neuroscience Multi-omics (NeMO) Archive (https://nemoarchive.org/).

Field-specific reporting

Please select the one below that is the best fit for your research. If you are not sure, read the appropriate sections before making your selection.

☒ Life sciences ☐ Behavioural & social sciences ☐ Ecological, evolutionary & environmental sciences

For a reference copy of the document with all sections, see nature.com/documents/nr-reporting-summary-flat.pdf

Life sciences study design

All studies must disclose on these points even when the disclosure is negative.

Sample size	Sample size to achieve reliable sampling (probability > 10%) of even very rare cell types (~0.1% prevalence) was estimated from a prior study of mouse cerebellum (Saunders, Macosko et al, Cell 2018 and see Extended Data Fig. 1); multiple replicates for each sex were included to reduce the effects of inter-individual variation. For electrophysiology and cell imaging experiments, no statistical methods were used to predetermine sample size. Sample sizes were deemed sufficient due to stark differences in presence of spikelets as well as some basic electrical properties between MLI1 and MLI2, which aligned with an observed bimodal distribution of data.
Data exclusions	For transcriptomic data, pre-established exclusion criteria of a minimum of 500 UMI/nucleus were used, as this is a standard threshold used in previous studies. Nuclei with a lower number of UMIs were excluded from any analysis.
Replication	For electrophysiology and cell imaging experiments demonstrating physiological and gene expression differences between the MLI1 and MLI2 populations, experiments were performed independently in different mice (n=18 mice). When molecular identification after patch clamp was unsuccessful, 2 photon and electrophysiology data were included without a molecular label.<br><br>All other reported transcriptomic findings were consistent across biological replicates.
Randomization	No differential experimental treatments were applied to individuals or samples in this study.
Blinding	Blinding was not relevant in this study as no differential experimental treatments were applied.

Reporting for specific materials, systems and methods

We require information from authors about some types of materials, experimental systems and methods used in many studies. Here, indicate whether each material, system or method listed is relevant to your study. If you are not sure if a list item applies to your research, read the appropriate section before selecting a response.

Materials & experimental systems

n/a	Involved in the study	
☒	☐	Antibodies
☒	☐	Eukaryotic cell lines
☒	☐	Palaeontology
☐	☒	Animals and other organisms
☐	☒	Human research participants
☒	☐	Clinical data

Methods

n/a	Involved in the study	
☒	☐	ChIP-seq
☒	☐	Flow cytometry
☒	☐	MRI-based neuroimaging

Animals and other organisms

Policy information about studies involving animals; ARRIVE guidelines recommended for reporting animal research

Laboratory animals	Mouse transcriptomic data was generated from 2 adult female and 4 adult male mice (60 days old; C57BL/6J, Jackson Labs). Mice were housed in a 12:12 light-dark cycle with ad libitum access to food and water.
Wild animals	No wild animals were used in the study.
Field-collected samples	No field-collected samples were used in the study.
Ethics oversight	All procedures involving animals at the Broad Institute were conducted in accordance with the US National Institutes of Health Guide for the Care and Use of Laboratory Animals under protocol number 0120-09-16.

Note that full information on the approval of the study protocol must also be provided in the manuscript.

Human research participants

Policy information about studies involving human research participants

Population characteristics	*Describe the covariate-relevant population characteristics of the human research participants (e.g. age, gender, genotypic information, past and current diagnosis and treatment categories). If you filled out the behavioural & social sciences study design questions and have nothing to add here, write "See above."*
Recruitment	*Describe how participants were recruited. Outline any potential self-selection bias or other biases that may be present and how these are likely to impact results.*
Ethics oversight	Human cerebellum tissue assayed was obtained from the NIH Brain and Tissue Repository of California, through the NIH NeuroBioBank. The tissue was received without identifiable information, and did not meet the definition of human subjects research (project # NHSR-4235).

Note that full information on the approval of the study protocol must also be provided in the manuscript.
